# Abrupt Decline in Tuberculosis among Foreign-Born Persons in the United States

**DOI:** 10.1371/journal.pone.0147353

**Published:** 2016-02-10

**Authors:** Brian J. Baker, Carla A. Winston, Yecai Liu, Anne Marie France, Kevin P. Cain

**Affiliations:** 1 Division of Tuberculosis Elimination, U.S. Centers for Disease Control and Prevention, Atlanta, Georgia, United States of America; 2 Division of Global Migration and Quarantine, U.S. Centers for Disease Control and Prevention, Atlanta, Georgia, United States of America; 3 Division of Tuberculosis Elimination, U.S. Centers for Disease Control and Prevention, Kisumu, Kenya; California Department of Public Health, UNITED STATES

## Abstract

While the number of reported tuberculosis (TB) cases in the United States has declined over the past two decades, TB morbidity among foreign-born persons has remained persistently elevated. A recent unexpected decline in reported TB cases among foreign-born persons beginning in 2007 provided an opportunity to examine contributing factors and inform future TB control strategies. We investigated the relative influence of three factors on the decline: 1) changes in the size of the foreign-born population through immigration and emigration, 2) changes in distribution of country of origin among foreign-born persons, and 3) changes in the TB case rates among foreign-born subpopulations. Using data from the U.S. National Tuberculosis Surveillance System and the American Community Survey, we examined TB case counts, TB case rates, and population estimates, stratified by years since U.S. entry and country of origin. Regression modeling was used to assess statistically significant changes in trend. Among foreign-born recent entrants (<3 years since U.S. entry), we found a 39.5% decline (-1,013 cases) beginning in 2007 (P<0.05 compared to 2000–2007) and ending in 2011 (P<0.05 compared to 2011–2014). Among recent entrants from Mexico, 80.7% of the decline was attributable to a decrease in population, while the declines among recent entrants from the Philippines, India, Vietnam, and China were almost exclusively (95.5%–100%) the result of decreases in TB case rates. Among foreign-born non-recent entrants (≥3 years since U.S. entry), we found an 8.9% decline (-443 cases) that resulted entirely (100%) from a decrease in the TB case rate. Both recent and non-recent entrants contributed to the decline in TB cases; factors contributing to the decline among recent entrants varied by country of origin. Strategies that impact both recent and non-recent entrants (e.g., investment in overseas TB control) as well as those that focus on non-recent entrants (e.g., expanded targeted testing of high-risk subgroups among non-recent entrants) will be necessary to achieve further declines in TB morbidity among foreign-born persons.

## Introduction

Tuberculosis (TB) remains an enormous public health challenge globally, with 9.6 million new cases and 1.5 million deaths each year [1]. Meanwhile, the United States has experienced a steady decline in TB cases over the past 2 decades, with a record low number of TB cases in 2014 [2]. This decline has predominately been among U.S.-born persons; the number of cases among foreign-born persons has remained relatively constant, and the TB case rate among the foreign-born population was 13.4 times greater than that of the U.S.-born population in 2014 [2]. A recent model estimated that TB elimination in the United States will not be achieved within the 21st century, partly because of a persistently elevated rate of TB among foreign-born persons [3].

Recent analyses revealed an unexpected decline in cases among foreign-born persons in the United States that began in 2007 [4, 5]. We investigated the relative influence of three factors on this decline: changes in the size of the foreign-born population through immigration and emigration, changes in the distribution of country of origin among foreign-born persons, and changes in the TB case rates among specific foreign-born subpopulations. We hypothesized that the decline among foreign-born persons was primarily because of a decreased rate of TB among recent entrants into the United States, a decreased number of foreign-born persons living in the United States (particularly recent entrants), and to a lesser extent, a decrease in transmission among foreign-born persons in the United States secondary to the lower number of cases due to the former two reasons.

## Methods

### Study Population

The study population included all foreign-born persons in the United States during 2000–2014. On the basis of preliminary findings, analyses of the decline focused on the period 2007–2011. Verified cases of reported TB were from the U.S. National Tuberculosis Surveillance System, which includes both laboratory-confirmed cases and clinical cases meeting pre-specified criteria [6]. Cases were classified by the patient’s country of origin and years since U.S. entry. Cases with unknown data regarding the patient’s country of origin or years since U.S. entry were excluded from the analysis. Genotyping data were from the U.S. National Genotyping Service [7]. Population estimates were derived from the American Community Survey 1-year estimates, an ongoing statistical survey that samples approximately 1.9 million households annually [8], and has been utilized for previous studies of TB case rates among foreign-born persons in the United States [9–11]. Individuals are included in the American Community Survey regardless of immigration status [8]. Data from the U.S. Department of Homeland Security [12] and CDC’s Electronic Disease Notification system [13] were used to estimate the number persons who entered into the United States in a given year, stratified by visa type and country of origin.

### Data Analysis

Joinpoint regression software, version 4.2.0.2 (National Cancer Institute, Bethesda, Maryland) was used to identify statistically significant changes in trend among TB case counts [14, 15]. Annual TB case rates were calculated by using case counts and population estimates for each year and were expressed as the number of cases per 100,000 persons. Detailed methods used to calculate rates stratified by years since U.S. entry have been described elsewhere [9–11].

Previous studies have established the high morbidity and elevated TB case rates among foreign-born persons during the first few years after entry into the United States [9–11, 16, 17].

To provide greater detail on these first few years, we divided foreign-born persons into the following groups based upon time since U.S. entry: <1 year, ≥1 to <2 years, ≥2 to <3 years, ≥3 to <6 years, and ≥6 years. We then concentrated further subpopulation analyses on recent entrants, defined as foreign-born persons <3 years since U.S. entry, as this represents the population most affected by changes in immigration and emigration [8, 18, 19]. We examined TB cases among recent entrants from the top five countries accounting for the largest number of cases diagnosed in the United States (i.e., Mexico, Philippines, India, Vietnam, and China) [2]. After excluding cases from the top five countries of origin, we categorized the remaining cases of TB among recent entrants according to the TB incidence in the country of origin as follows: low, <15 cases/100,000 persons; medium, 15–99 cases/100,000 persons; or high incidence, ≥100/100,000 persons [1].

To evaluate potential causes of the decline among each foreign-born subpopulation, we estimated the proportion of the case count decrease resulting from changes in the TB case rate versus population shifts using Formula 1 and Formula 2 below. If there was an increase in population from 2007 to 2011, then 100% of the case count decline was assumed to be the result of changes in TB case rate.

**Formula 1.** Percent of case count decline resulting from TB case rate change =

$$\frac{P^{2007}\;*\;(R^{2011}-R^{2007})}{P^{2007}\;*\;(R^{2011}-R^{2007})+R^{2007}\;*\;(P^{2011}-P^{2007})}$$

R^2007^ = TB case rate in 2007; R^2011^ = TB case rate in 2011; P^2007^ = Population in 2007; P^2011^ = Population in 2011

**Formula 2.** Percent of case count decline resulting from population change =

$$\frac{R^{2007}\;*\;(P^{2011}-P^{2007})}{P^{2007}\;*\;(R^{2011}-R^{2007})+R^{2007}\;*(P^{2011}-P^{2007})}$$

R^2007^ = TB case rate in 2007; R^2011^ = TB case rate in 2011; P^2007^ = Population in 2007; P^2011^ = Population in 2011

In 2007, CDC updated requirements for overseas TB screening of refugees and persons applying for permanent resident visas to the United States [20, 21]. Among the most important changes was the introduction of routine mycobacterial culture (in addition to the already required sputum-smear microscopy). Those applying for other visa types (e.g., students, exchange visitors, temporary workers, tourists, and business travelers) are not routinely required to undergo overseas TB screening, and those who enter the United States without a visa (e.g., undocumented persons) are not subject to overseas screening. We used data from the U.S. Department of Homeland Security and CDC’s Electronic Disease Notification system to assess the scope of overseas TB screening by estimating the number of persons each year who arrived as refugees or permanent resident applicants. We then used American Community Survey data to estimate the overall population of new entrants, defined as the population that entered the United States within the past year, regardless of visa type (including persons without a visa). For each country, we calculated the proportion of the new entrant population who passed through culture-based overseas TB screening during 2007–2011. For countries where culture-based overseas screening only occurred for part of a year [22], we assumed that the number of refugees and permanent resident applicants was evenly distributed by month and prorated the number screened accordingly.

Finally, focusing on cases among recent entrants (<3 years since U.S. entry), we estimated the number of cases associated with likely recent transmission in the United States. We used a methodology verified against a field-based standard [23], with two alterations. First, we included cases diagnosed <100 days after U.S. entry to avoid underestimating cases possibly due to local transmission shortly after arrival. Second, a case attributed to recent transmission must have had a U.S. entry date before the diagnosis date of the possible source case. The U.S. National Genotyping Service introduced universal 24-locus mycobacterial interspersed repetitive unit–variable number tandem repeat genotyping in 2009 [7]. To ensure a 2-year time interval for a potential source case to be identified, the transmission component of the analysis was limited to cases counted during January 1–September 30, 2011, in the U.S. National Tuberculosis Surveillance System. Cases diagnosed during October 1–December 31, 2011, were excluded to allow an additional 3-month period for belated identification of potential source cases. A given case was classified as due to recent transmission if at least one plausible source case was identified based upon clinical characteristics, genotyping data, and temporal and geographic proximity to the putative secondary case. In addition, we examined the reported origin (U.S.-born versus foreign-born) for each potential source case. Some secondary cases were found to have more than one potential source case; therefore, we generated low and high estimates of possible source cases by origin.

Aside from analyses of Joinpoint regression and changes in trend, all additional analyses were conducted using SAS, version 9.3 (SAS Institute, Inc., Cary, North Carolina). For all statistical tests, results were considered to be significant at P<0.05. CDC reviewed the proposed study and determined that approval by an institutional review board was not required because data were collected and analyzed for this project as part of routine TB surveillance; therefore, the project is not considered research involving human subjects.

## Results

### Trends in TB Case Counts, 2000–2014

**Fig 1:**
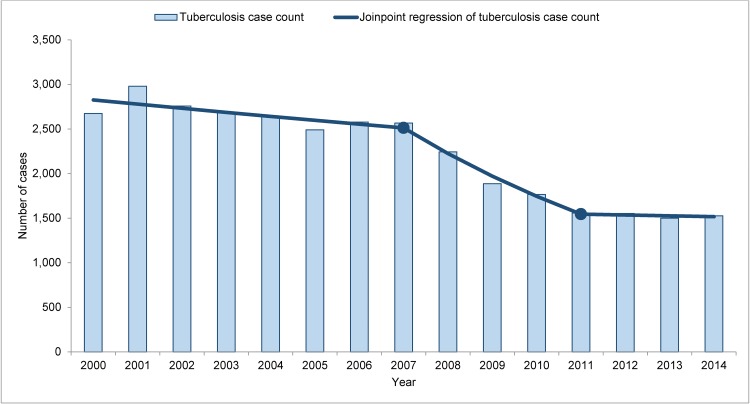
Tuberculosis case counts with Joinpoint regression among recent entrants, 2000–2014. Shaded bars indicate tuberculosis case counts among recent entrants (foreign-born persons with <3 years since U.S. entry). The solid line represents a Joinpoint regression of tuberculosis case counts. Solid circles represent statistically significant changes in trend in 2007 and 2011 (2000–2007 annual percent change, -1.7% [95% confidence interval {CI}, -3.5%, +0.2%); 2007–2011 annual percent change, -11.4% (95% CI -17.4%, -5.1%)]; 2011–2014 annual percent change, -0.6% [95% CI, -7.3%, +6.5%]).

There were 108,944 cases of TB reported among foreign-born persons in the United States during 2000–2014. Data were missing for country of origin or time since U.S. entry for 4,913 persons (4.5%), leaving 104,031 cases for analysis. TB cases among recent entrants (<3 years since U.S. entry) declined by 42.9% (-1,148 cases) during 2000–2014, with statistically significant changes in trend in 2007 and 2011 (Fig 1). In 2014, recent entrants accounted for 26.2% of all cases among foreign-born persons, compared with 37.0% in 2000.

### Time since U.S. Entry, 2007–2011

During 2007–2011, there was an overall decline of 1,456 cases (-19.3%) among all foreign-born persons (Table 1). Among recent entrants (<3 years since U.S. entry), there were decreases in case count (-39.5%, -1,013 cases), TB case rate (-30.8%), and population (-12.5%). Among non-recent entrants (≥3 years since U.S. entry), there was a decrease in case count (-8.9%, -443 cases) and TB case rate (-16.6%), but an increase in the non-recent entrant population (+9.1%).

**Table 1:** Changes in tuberculosis case counts, population estimates, and tuberculosis case rates among foreign-born persons in the United States, 2007 and 2011.

		Tuberculosis Case Count			Population Estimate^a^ (Thousands)			Tuberculosis Case Rate (per 100,000 Persons)		
		2007	2011	Relative Change (%)	2007	2011	Relative Change (%)	2007	2011	Relative Change (%)
Among all foreign born persons										
		7,529	6,073	-19.3	40,148	42,917	+6.9	18.8	14.2	-24.5
Recent versus non-recent entrants^b^										
	Recent	2,567	1,554	-39.5	4,152	3,632	-12.5	61.8	42.8	-30.8
	Non-recent	4,962	4,519	-8.9	35,996	39,285	+9.1	13.8	11.5	-16.6
Years since U.S. entry										
	<1	1,521	935	-38.5	1,449	1,318	-9.1	104.9	71.0	-32.4
	1 to <2	557	330	-40.8	1,369	1,175	-14.2	40.7	28.1	-31.0
	2 to <3	489	289	-40.9	1,334	1,140	-14.6	36.7	25.4	-30.8
	3 to <6	1,005	838	-16.6	4,016	3,661	-8.8	25.0	22.9	-8.5
	≥6	3,957	3,681	-7.0	31,979	35,624	+11.4	12.4	10.3	-16.5

^a^ Source: American Community Survey [8].

^b^ Recent entrants are foreign-born persons with <3 years since U.S. entry; non-recent entrants are foreign-born persons with ≥3 years since U.S. entry.

**Fig 2:**
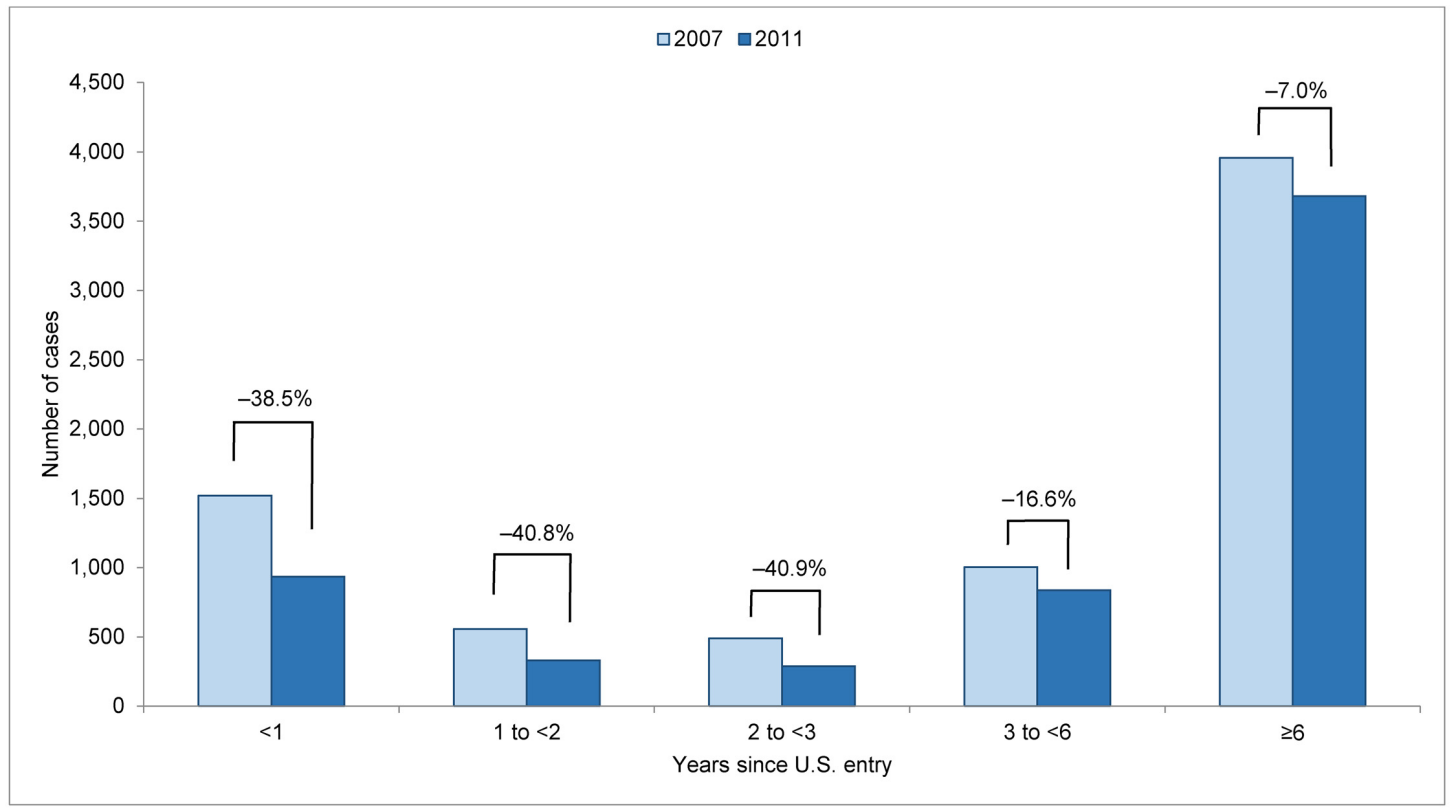
Tuberculosis case counts and percent decline among foreign-born persons by years since U.S. entry, 2007 and 2011. Light shaded bars represent tuberculosis case counts in 2007, and dark shaded bars represent tuberculosis case counts in 2011. Percentages indicate the percent decline in case count for each group during 2007–2011.

When examining more discrete groups by years since U.S. entry (Fig 2), case counts declined among all groups, with the smallest relative case count decline among foreign-born persons in the United States ≥6 years (-7.0%, -276 cases). TB case rates also declined among all foreign-born groups, regardless of time since U.S. entry (Table 1). Although the population of foreign-born persons with ≥6 years since U.S. entry increased (+11.4%), decreases in population were seen among all other groups.

### Recent Entrants: Top Five Countries of Origin, 2007–2011

**Fig 3:**
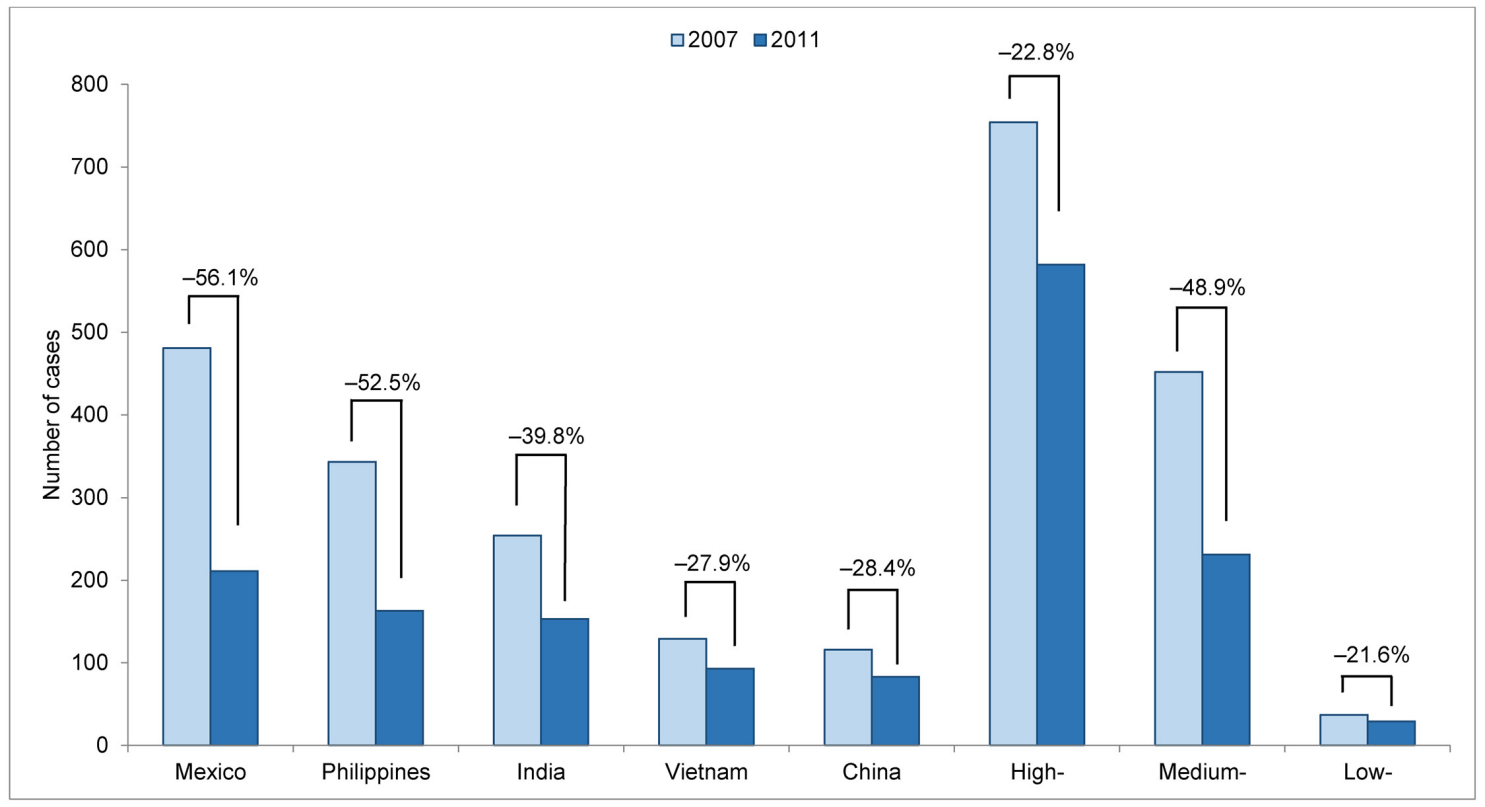
Tuberculosis case counts and percent decline among recent entrants, by country of origin or tuberculosis incidence in country of origin, 2007 and 2011. Light shaded bars represent tuberculosis case counts in 2007, and dark shaded bars represent tuberculosis case counts in 2011. Percentages indicate the percent decline in case count for each group during 2007–2011. Recent entrants are foreign-born persons with <3 years since U.S. entry. The top five countries of origin accounting for the greatest number of tuberculosis cases among foreign-born persons in the United States are listed; cases among persons from other countries are classified according to tuberculosis incidence in the country of origin as low (<15 cases/100,000 persons), medium (15–99 cases/100,000 persons), or high (≥100 cases/100,000 persons). Countries with unknown incidence rates were excluded. All incidence rates used to categorize countries of origin are from World Health Organization data [1] and expressed per 100,000 persons.

TB case counts among recent entrants from all of the top five countries of origin declined during 2007–2011 (Fig 3). The largest relative declines in case counts were among recent entrants from Mexico (-56.1%, -270 cases), the Philippines (-52.5%, -180 cases), and India (-39.8%, -101 cases).

When examining TB case rates, the greatest relative declines occurred among recent entrants from the Philippines (-51.3%), India (-47.3%), China (-47.0%), and Vietnam (-37.1%), whereas the smallest TB case rate decline was among Mexico-born recent entrants (-12.0%) (Table 2). The population of recent entrants from Mexico decreased (-50.1%), but the populations of recent entrants from China (+35.0%), Vietnam (+14.6%), and India (+14.3%) all increased, and the population of recent entrants from the Philippines remained largely unchanged (-2.4%).

**Table 2:** Changes in tuberculosis case counts, population estimates, and tuberculosis case rates among recent^a^ entrants, 2007 and 2011.

		Tuberculosis Case Count			Population Estimate^b^ (Thousands)			Tuberculosis Case Rate (per 100,000 Persons)		
		2007	2011	Relative Change (%)	2007	2011	Relative Change (%)	2007	2011	Relative Change (%)
County of origin, top five high morbidity^c^										
	Mexico	481	211	-56.1	1,219	608	-50.1	39.5	34.7	-12.0
	Philippines	343	163	-52.5	172	167	-2.4	199.9	97.3	-51.3
	India	254	153	-39.8	257	294	+14.3	98.7	52.0	-47.3
	Vietnam	129	93	-27.9	68	78	+14.6	188.5	118.6	-37.1
	China	116	83	-28.4	195	263	+35.0	59.5	31.5	-47.0
Incidence in countries of origin, excluding the top five^d^										
	High	754	582	-22.8	449	481	+7.0	167.5	119.1	-28.9
	Medium	452	231	-48.9	1,006	943	-6.3	44.9	23.7	-47.4
	Low	37	29	-21.6	681	690	+1.3	5.4	4.2	-22.6

^a^ Recent entrants are foreign-born persons with <3 years since U.S. entry.

^b^ Source: American Community Survey [8].

^c^ The five countries of origin accounting for the greatest number of tuberculosis cases in the United States. In 2011, the estimated TB incidences in the top five countries were: Mexico (21/100,000 persons), Philippines (303/100,000 persons), India (80/100,000 persons), Vietnam (180/100,000 persons), China (75/100,000 persons) [1].

^d^ Excluding cases from the top five countries of origin, we categorized the remaining cases of tuberculosis among recent entrants according to the tuberculosis incidence in the country of origin: low (<15 cases/100,000 persons), medium (15–99 cases/100,000 persons), or high (≥100 cases/100,000 persons) [1].

### Recent Entrants: Country of Origin, Categorized by TB Incidence Rate, 2007–2011

Excluding the top five countries of origin, case count declines occurred among recent entrants from all groups, categorized by TB incidence rate, with the largest relative case count decline from medium-incidence countries (-48.9%, -221 cases) (Fig 3). Recent entrants from all incidence groups also experienced declines in TB case rate (Table 2). The populations of recent entrants from low- (+1.3%) and high-incidence (+7.0%) countries both increased, but the population of recent entrants from medium-incidence countries decreased (-6.3%).

### Factors Contributing to the Decline, 2007–2011

Overall, 69.6% of the case count decline was among recent entrants (1,013 of 1,456 cases). Among recent entrants, an estimated 71.1% of the decline was from a decrease in the TB case rate, and the remaining 28.9% was the result of a decrease in the recent entrant population. When stratified by country of origin, 100% of the respective case count declines among recent entrants from China, India, and Vietnam (and 95.5% from the Philippines) were found to be the result of decreases in TB case rate. For recent entrants from Mexico, 80.7% of the case count decline was attributable to the decreased population, and 19.3% was a result of a decrease in TB case rate.

Non-recent entrants (≥3 years since U.S. entry) accounted for 30.4% of the overall foreign-born case count decline (443 of 1,456 cases). Among non-recent entrants, 100% of the case count decline was the result of a decrease in TB case rate—the non-recent entrant population increased during the study period.

### Overseas Culture-Based TB Screening by Country of Origin, 2007–2011

During 2007–2011, the proportion of the new entrant population (<1 year since U.S. entry) with overseas culture-based TB screening varied by country of origin (Table 3). India, China, and Mexico had the lowest proportion of new entrants who received overseas TB screening (5.4%, 20.3%, and 23.7%, respectively), despite comprising the largest new entrant populations. The majority of the new entrant population from the Philippines (51.7%) and Vietnam (72.6%) received overseas culture-based TB screening during 2007–2011.

**Table 3:** Proportion of new entrant population^a^ with overseas culture-based tuberculosis screening, by country of origin, 2007–2011.

Country of Origin	Implementation Date^b^ for Overseas Culture-Based Screening	New Entrant Population Estimate^a^ (Thousands)	Number of New Entrants with Overseas Culture-Based Screening^c^ (Thousands)	Proportion of New Entrant Population with Overseas Culture-Based Screening (%)
Mexico	October 2007	1,315	311	23.7
Philippines	October 2007	290	150	51.7
India	October 2010	571	31	5.4
Vietnam	February 2008	135	98	72.6
China	July 2009	441	89	20.3

^a^ Foreign-born persons living in the United States for <1 year, regardless of visa status or mode of entry. Source: American Community Survey [8].

^b^ These dates represent the transition from smear-based to culture-based overseas screening. Source: Centers for Disease Control and Prevention [22].

^c^ Only refugees or persons applying for permanent resident visas must undergo overseas tuberculosis screening. Those applying for other visa types (e.g., students, exchange visitors, temporary workers, tourists, and business travelers) and those who enter the United States without a visa (e.g., undocumented persons) are not required to receive overseas screening. Sources: U.S. Department of Homeland Security [12] and CDC’s Electronic Disease Notification system [13].

World Health Organization estimates [1] from 2007 and 2011 reveal declines in estimated TB incidence in China (-12.8%), India (-10.4%), Vietnam (-10.1%), and the Philippines (-6.8%). In contrast, Mexico had a slight increase in estimated TB incidence (+5.0%) (Table 4).

**Table 4:** Changes in Estimated Overseas Tuberculosis Incidence for the Top Five Countries of Origin^a^, 2007 and 2011.

		Estimated Tuberculosis Incidence^b^ (per 100,000 Persons)		Percent Change (%)
		2007	2011	
Country of origin				
	Mexico	20	21	+5.0
	Philippines	325	303	-6.8
	India	201	180	-10.4
	Vietnam	168	151	-10.1
	China	86	75	-12.8

^a^ The five countries of origin accounting for the greatest number of tuberculosis cases among foreign-born persons in the United States.

^b^ Source: World Health Organization [1].

### Transmission of TB among Recent Entrants in the United States, 2011

During January 1–September 30, 2011, of the 1,039 cases among recent entrants, 772 (74.3%) had at least one positive culture result (Table 5). Among the 720 (93.3%) cases with a known TB genotype, 30 (4.2%) were likely due to recent transmission and had at least one potential source case identified. We estimated that 12–16 of the 720 cases (1.7%–2.2%) were likely due to transmission from a recent entrant with infectious TB.

**Table 5:** Transmission of *Mycobacterium tuberculosis* among recent^a^ entrants, 2011^b^.

		Number or Estimated Range^c^	%
Cases among recent entrants		1,039	
With at least one positive culture result		772/1,039	74.3
With a genotype result		720/772	93.3
Due to recent transmission^d^		30/720	4.2
By origin of source case			
	Foreign-born, recent entrant^a^ source case	12–16/720	1.7–2.2
	Foreign born, non-recent^a^ entrant source case	11–14/720	1.5–1.9
	U.S.-born source case	3–4/720	0.4–0.6

^a^ Recent entrants are foreign-born persons with <3 years since U.S. entry; non-recent entrants are foreign-born persons with ≥3 years since U.S. entry.

^b^ Cases counted during January 1–September 30, 2011, in the U.S. National Tuberculosis Surveillance System; cases from October 1–December 31, 2011, were excluded to allow an additional 3-month period for belated identification of potential source cases.

^c^ Range reflects low–high estimates. Low estimates include only scenarios where all potential source cases are from a given group (e.g., foreign-born recent entrants). High estimates include scenarios where at least one potential source case is from a given group.

^d^ A given case was classified as due to recent transmission if at least one plausible source case was identified based upon clinical characteristics, genotyping data, and temporal and geographic proximity to the putative secondary case.

## Discussion

We found an overall decline of 1,456 cases (-19.3%) among foreign-born persons during 2007–2011. All foreign-born subpopulations, regardless of time since U.S. entry, experienced declines in both TB case count and case rate.

We had hypothesized that the decline in TB cases among foreign-born persons was primarily the result of a decreased TB case rate among recent entrants (<3 years since U.S. entry) and a decreased number of foreign-born persons living in the United States (particularly recent entrants). Our overall findings support these hypotheses. Specifically, we determined that the majority (69.6%) of the case count decline was among recent entrants; 71.1% of the case count decline among recent entrants was the result of decreased TB case rates, and 28.9% was from a population decrease.

When we examined contributing factors to the decline among subpopulations of recent entrants, we identified substantial heterogeneity by country of origin. Among Mexico-born recent entrants, 80.7% of the case count decline was the result of a population decrease, and only 19.3% was from a case rate decline, consistent with previously reported results [11]. These findings imply that if immigration from Mexico were to markedly increase, the number of TB cases among Mexico-born recent entrants would also be expected to increase. In contrast, the case count decline among recent entrants among each of the other top five countries of origin was almost entirely the result of a case rate decline and therefore might be less affected by future immigration trends. These observations become particularly important in the context of ongoing demographic shifts among foreign-born persons in the United States [18, 19, 24].

At least four major factors could explain the observed declines in TB case rates among recent entrants: overseas culture-based TB screening, declining TB morbidity in the countries of origin, changing composition of recent entrants (e.g., a disproportionate decrease in recent entrants from high-incidence countries), and decreased transmission in the United States among recent entrants.

Previous studies have assessed the benefits of culture-based overseas TB screening [25–29], and a recent analysis specifically explored the contribution of culture-based overseas screening to the decline in foreign-born TB incidence in the United States [30]. We found that the proportion of recent entrants who received overseas culture-based TB screening varied greatly by country of origin. During 2007–2011, only 23.7% of the new entrant population from Mexico passed through overseas culture-based TB screening, in contrast with 72.6% of new entrant population from Vietnam. Because we found a stable TB case rate among recent entrants from Mexico and a large case rate decline among recent entrants from Vietnam, these data are consistent with a contribution to TB case reduction from overseas culture-based TB screening, particularly in countries where the screening covered a high percentage of the new entrant population.

However, like Mexico, a smaller proportion of the new entrant population from India (5.4%) and China (20.3%) passed through overseas culture-based TB screening during 2007–2011, and yet recent entrants from India and China also experienced substantial declines in TB case rates. These data demonstrate that, although overseas culture-based TB screening likely contributed to the TB case rate decline for specific countries of origin (e.g., Vietnam and the Philippines), other factors likely played a more prominent role in the TB case rate decline among recent entrants from other countries of origin (e.g., Mexico, India, and China). Prior studies have explored the potential impact of extending overseas TB screening to additional visa categories [28, 31] as well as testing and treating latent TB infection among refugees overseas [32]. While such programs might further reduce TB morbidity in the United States, these efforts would likely have a limited impact on persons originating from Mexico, the vast majority of whom enter the United States with a short-term visa or without a visa altogether [12, 19].

The second factor potentially contributing to the decline in TB case rates among recent entrants is decreased TB incidence in each of the top five countries of origin. From 2007–2011, there were declines in estimated TB incidence in China, India, Vietnam, and the Philippines, but a relatively stable estimated TB incidence in Mexico. Based upon these data, it is possible that declining morbidity in foreign countries contributed to the declining case rates seen among recent entrants from China, Vietnam, India, and the Philippines. However, as recent entrants may not be representative of the general population in the country of origin, the precise contribution of decreasing TB incidence overseas to the decline in TB cases in the United States remains uncertain.

The third factor that could explain the decrease in TB case rates is the changing composition of the recent entrant population. Excluding the top five countries of origin, the population of recent entrants from medium-incidence countries decreased (-6.3%) during the study period, while the population of recent entrants from low-incidence countries remained largely unchanged (+1.3%), and the population of recent entrants from high-incidence countries increased (+7.0%). There was a large decline in the recent entrant population from Mexico, but the recent entrant populations from Vietnam and the Philippines (high-incidence countries) as well as China and India (medium-incidence countries) all experienced increases or no change (Philippines) during 2007–2011. These findings indicate that the changing composition of recent entrants did not substantially contribute to the overall decline in TB case rates among recent entrants.

The fourth factor that could explain a decrease in TB case rates is less TB transmission among recent entrants. However, we found that a limited proportion of cases among recent entrants were likely due to recent transmission (4.2%), and an even smaller proportion (1.7%–2.2%) were likely due to transmission from a recent entrant source case. These findings are consistent with prior studies that established relatively low rates of transmission among foreign-born persons in the United States [33, 34]. Although data on transmission was limited to 2011 (and we could not assess trends over time), the extremely small proportion of cases associated with transmission make it unlikely that a change in transmission was a major contributor to the decline.

A substantial portion of the overall case count decline (30.4%) was among non-recent entrants (≥3 year since U.S. entry), and was therefore not accounted for by our hypotheses. Importantly, the case count decline among non-recent entrants was exclusively the result of a decrease in TB case rates. These decreasing rates might reflect a birth-cohort effect from a lower prevalence of latent TB infection among successive cohorts of immigrants [35]. This is of particular interest, as we must consider that a substantial proportion of the decline observed might be the result of global TB control efforts in foreign countries during the past several decades—a reminder of the importance of domestic returns from investing in TB control overseas [36]. Another factor that could explain the observed decline among non-recent entrants is improved TB control efforts in the United States through targeted testing and treatment of latent TB infection. However, targeted testing has not previously focused on non-recent entrants; therefore, this explanation seems less likely [37].

The substantial decline in TB case rate among non-recent entrants was an unexpected finding, and has implications for future TB morbidity trends in the United States. Despite a substantial decline in TB case rate (-16.6%), an increase in the size of the non-recent entrant population (+9.1%) dampened the reduction in TB cases. Non-recent entrants accounted for 73.8% of all cases among foreign-born persons in 2014, and if current trends continue, can be expected to be the dominant driver of future TB epidemiology in the United States.

Because of the enormous size of the non-recent entrant population (an estimated 41.2 million in 2014) [8], domestic efforts to reduce the TB case rate among this group (i.e., targeted testing and treatment of latent TB infection) could have a profound impact on TB morbidity as a whole in the United States. Although U.S. national guidelines recommend prioritizing testing of foreign-born persons who have been in the United States for <5 years [37], recent evidence suggests that rates of reactivation remain persistently elevated for years after arrival [17, 38]. At the same time, providing targeted testing and treatment to the entirety of the foreign-born population might not be feasible, given current health system resources [39, 40]. Therefore, there is an urgent need to identify a practical, prioritized approach to latent TB infection among foreign-born persons [41]. Studies that examine the cost-effectiveness of targeting foreign-born persons with specific risk factors for reactivation [42] could be used to focus testing and treatment of latent TB infection among this large and growing population.

One of the primary goals of TB surveillance is to understand trends in morbidity and to shape public health policies. However, because of shifts in immigration and changes in the distribution of TB cases among the foreign-born population (especially with respect to duration of time since U.S. entry), the standard approach to interpreting TB case counts and overall TB case rates might not be adequate. For example, if immigration increases in the future, this might result in an increase in foreign-born TB cases and be mistakenly interpreted as a failure of TB control efforts. Conversely, a further reduction in the TB case rate among non-recent entrants might result in an overall decline in cases among foreign-born persons, which might mask a simultaneous increase among recent entrants.

Our analyses have several limitations. First, there is statistical variability in the timing of the Joinpoint regression flexions, indicating the timing of the decline, and as a result, 2007–2011 is an estimate. Second, in the transmission analysis, we included persons diagnosed with TB <100 days after arrival, and therefore, might have misclassified cases as due to recent transmission in the United States that actually were due to transmission prior to U.S. entry. Third, as we were only able to assess transmission for TB cases diagnosed in 2011, we were unable to specifically evaluate changes in transmission during 2007–2011. Fourth, while the American Community Survey does not exclude persons on the basis of immigration status, it is possible that some proportion of undocumented persons are missed. Fifth, although a small proportion (4.5%) of overall cases had unknown years since U.S. entry and/or country of origin, these cases may differ from the overall study population. Sixth, persons may have passed through Mexico en route from another Latin American country to the United States, and could have been misclassified as Mexico-born.

Overall, we found that during 2007–2011, there was an abrupt decline in reported TB cases among foreign-born persons living in the United States, and this decline was evident among both recent and non-recent entrants. Among recent entrants, the decline was associated with a decrease in TB case rate as well as a decrease in population, but this varied by country of origin. In addition, non-recent entrants also substantially contributed to the decline, exclusively as a result of a decreasing TB case rate. Future TB morbidity trends among recent entrants may be closely linked to patterns of immigration and emigration. Strategies that impact both recent and non-recent entrants (e.g., investment in overseas TB control) as well as those that focus on non-recent entrants (e.g., targeted testing of high-risk subpopulations among non-recent entrants) will be necessary to achieve further declines in TB morbidity among foreign-born persons.
